# Bilaterally symmetric axes with rhizoids composed the rooting structure of the common ancestor of vascular plants

**DOI:** 10.1098/rstb.2017.0042

**Published:** 2017-12-18

**Authors:** Alexander J. Hetherington, Liam Dolan

**Affiliations:** Department of Plant Sciences, University of Oxford, South Parks Road, Oxford OX1 3RB, UK

**Keywords:** Rhynie chert, root, root evolution, rhizoidal sporophyte axes, palaeobotany

## Abstract

There are two general types of rooting systems in extant land plants: gametophyte rhizoids and sporophyte root axes. These structures carry out the rooting function in the free-living stage of almost all land plant gametophytes and sporophytes, respectively. Extant vascular plants develop a dominant, free-living sporophyte on which roots form, with the exception of a small number of taxa that have secondarily lost roots. However, fossil evidence indicates that early vascular plants did not develop sporophyte roots. We propose that the common ancestor of vascular plants developed a unique rooting system—rhizoidal sporophyte axes. Here we present a synthesis and reinterpretation of the rootless sporophytes of *Horneophyton lignieri*, *Aglaophyton majus*, *Rhynia gwynne-vaughanii* and *Nothia aphylla* preserved in the Rhynie chert. We show that the sporophyte rooting structures of all four plants comprised regions of plagiotropic (horizontal) axes that developed unicellular rhizoids on their underside. These regions of axes with rhizoids developed bilateral symmetry making them distinct from the other regions which were radially symmetrical. We hypothesize that rhizoidal sporophyte axes constituted the rooting structures in the common ancestor of vascular plants because the phylogenetic positions of these plants span the origin of the vascular lineage.

This article is part of a discussion meeting issue ‘The Rhynie cherts: our earliest terrestrial ecosystem revisited’.

## 1. Introduction

Structures that carried out rooting functions were one of a suite of adaptations that evolved in plants during or soon after the colonization of land [1–3]. They provided the functions of anchorage, nutrient uptake and water absorption that are essential for the growth and development of land plants [4–6]. Rooting structures increased in size and complexity during the explosion of morphological diversity that occurred as plants radiated and spread from damper to drier regions of the continental surfaces during the Palaeozoic [4–8]. This dramatically affected the Earth system by impacting both the carbon and hydrological cycles. The evolution of rooting systems and their symbionts [9–11] modulated the carbon cycle by enhancing the weathering of silicate rocks, increasing carbon burial and consequently reducing atmospheric CO_2_ levels [5,12,13]. Moreover, rooting structures further enhanced carbon burial by contributing to the formation of the first complex soils [8], the largest carbon sink after the oceans today [14]. The evolution of roots altered the hydrological cycle by transforming the morphology of rivers from braided to meandering systems and in doing so increased the stability of terrestrial sediments [15,16]. The evolution of roots and their diversification, therefore, had dramatic impacts on the biotic and abiotic processes in the Earth system.

The diversity of rooting structures in extant land plants can be grouped into two broad categories [1,6]. (1) Unicellular or multicellular tip-growing, tubular structures called rhizoids carry out rooting functions in the gametophytes (haploid, multicellular phase of the life cycle) in extant non-vascular plants (liverworts, hornworts and mosses) [1]. Similar rhizoid systems develop in the gametophytes of lycophytes (clubmosses, spike mosses and quillworts) and monilophytes (ferns and horsetails) [1]. (2) Rooting structures of extant vascular plant (tracheophyte) sporophytes (diploid, multicellular phase of life cycle) comprise specialized rooting axes that develop from meristems covered with a root cap [5]. A defining feature of roots is the cap-covered meristem. Roots often, though not always, develop endogenonusly (with the exception of some lycophytes, see [17]), exhibit positive gravitropism (although roots are not solely positively gravitropic [18]), and form an endodermis (although an endodermis is not a distinguishing feature of roots in some lycophytes [5]). Most roots develop unicellular tubular epidermal outgrowths, called root hairs [1]. Roots may branch—dichotomously in lycophytes [17] or subapically in other vascular plants [19]—to form networks that penetrate the soil and form specialized symbiotic structures (mycorrhizae or nodules) [10,11]. These two types of rooting structures carry out rooting function in plants with free-living gametophytes and sporophytes respectively.

**Figure 1.**
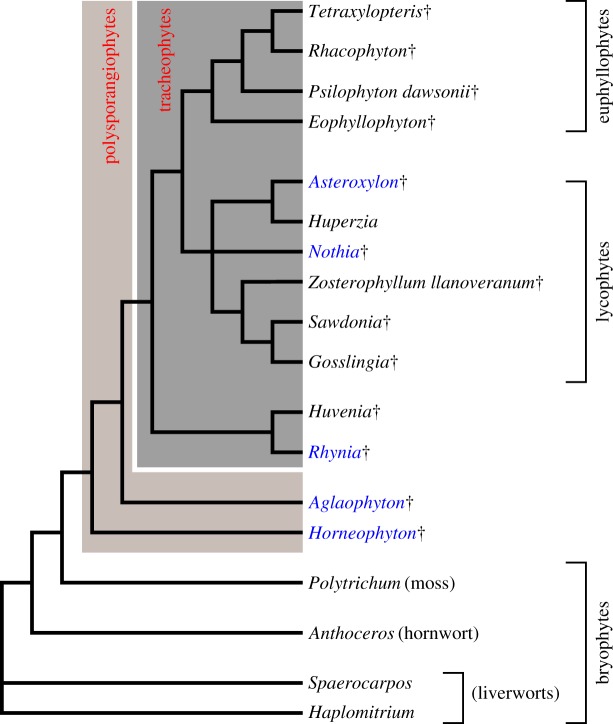
Cladogram of relationships among land plants after reference [2]. The cladogram is a reproduction of the result of analysis 4.2 from reference [2]. †Extinct taxa. Plants preserved in the Rhynie chert highlighted in blue. Shading highlights both the polysporangiophytes and vascular plants (tracheophytes).

The fossil record supports the hypothesis that the common ancestor of vascular plants comprised free-living gametophyte and sporophyte generations [2,20–22]. Gametophytic rhizoids also carried out rooting functions in free-living gametophytes of early vascular plants such as *Remyophyton delicatum* (the gametophyte of *Rhynia gwynne-vaughanii*) which is preserved in the Rhynie chert [23,24]. However, the sporophyte generation of these plants were rootless and cladistic analysis of extant and extinct land plants supports the hypothesis that the common ancestor of vascular plants was also rootless [2,5,25,26]. Four sporophytes preserved in the Rhynie chert—*Horneophyton lignieri*, *Aglaophyton majus*, *R. gwynne-vaughanii* and *Nothia aphylla*—occupy a key phylogenetic position for investigating the nature of rooting structures in the common ancestor of vascular plants. These species span the origin of the vascular plant lineage (figure 1) [2]. By describing the rooting structures of these four plants—the two protracheophytes, *H. lignieri* and *A. majus*, the basal vascular plant *R. gwynne-vaughanii* and the tentative lycophyte *N. aphylla* (see discussion of the uncertain phylogenetic placement of *N. aphylla* in [2,27,28])—we can investigate the structure of the sporophyte rooting system present in the common ancestor of vascular plants. Here, we present a synthesis and reinterpretation of the sporophyte rooting structures of *H. lignieri*, *A. majus*, *R. gwynne-vaughanii* and *N. aphylla* preserved in the Rhynie chert. Using data collected over the last century and new measurements made from these fossils, we propose that rhizoidal sporophyte axes that carried out the rooting function represent a third major type of land plant rooting system.

## 2. Material and methods

**Figure 2.**
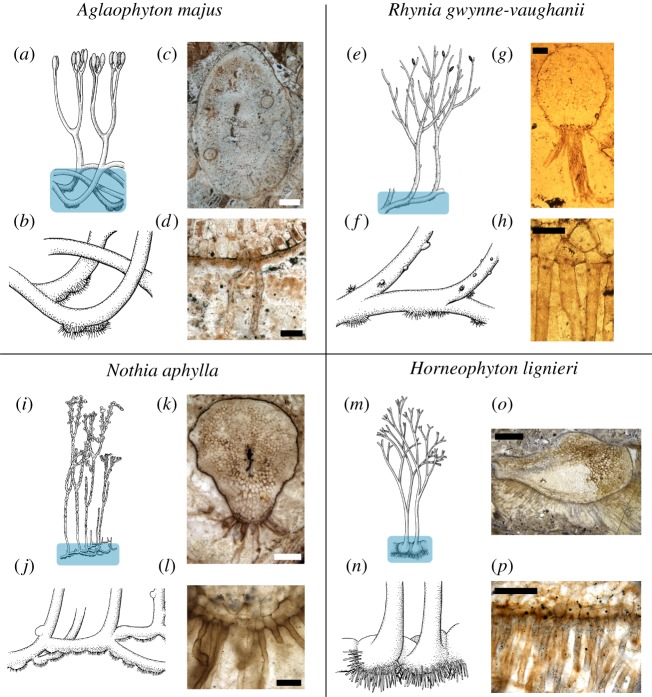
Rhizoidal sporophyte axes of *A. majus* (*a*–*d*), *R. gwynne-vaughanii* (*e*–*h*), *N. aphylla* (*i*–*l*) and *H. lignieri* (*m*–*p*). (*a*) Anatomical reconstruction of *A. majus* after [17], (*b*) enlarged reconstruction of the rhizoidal sporophyte axes of *A. majus* (drawn by Rosemary Wise based on [2]), (*c*) transverse section through the rhizoidal sporophyte axis of *A. majus* showing rhizoids developing from the underside of this axis, (*d*) higher magnification image of (*c*) showing unicellular rhizoids developing from the epidermis. (*e*) Anatomical reconstruction of *R. gwynne-vaughanii* after [2], (*f*) enlarged reconstruction of the rhizoidal sporophyte axes of *R. gwynne-vaughanii* (drawn by Mrs R. Wise based on [2]), (*g*) transverse section through the rhizoidal sporophyte axis of *R. gwynne-vaughanii* showing rhizoids developing from the underside of this axis, (*h*) higher magnification image of (*g*) showing unicellular rhizoids developing from the epidermis. (*i*) Anatomical reconstruction of *N. aphylla* after [28], (*j*) enlarged reconstruction of the rhizoidal sporophyte axes of *N. aphylla* (drawn by Mrs R. Wise based on [28]), (*k*) transverse section through the rhizoidal sporophyte axis of *N. aphylla* showing rhizoids developing from the underside of this axis, (*l*) higher magnification image of (*k*) showing unicellular rhizoids developing from the epidermis. (*m*) Anatomical reconstruction of *H. lignieri* after [2], (*n*) enlarged reconstruction of rhizoidal sporophyte axes of *H. lignieri* (drawn by Mrs R. Wise based on [2]), (*o*) transverse section through the rhizoidal sporophyte axis of *H. lignieri* showing large numbers of rhizoids developing from the underside of this axis, (*p*) higher magnification image of (*o*) showing unicellular rhizoids developing from the epidermis. Scale bars, 500 µm (*k*,*o*,*c*), 200 µm (*g*,*p*), 100 µm (*d*), 50 µm (*h*, *l*). (*c*,*d*) Thin section GLAHM 2396 Kidston Collection, Hunterian Museum, University of Glasgow. (*g*,*h*) AGL. Block 22. (courtesy of Professor Dianne Edwards). (*k*,*l*) Slide P 2868, (courtesy of Professor Hans Kerp). (*o*,*p*) Thin section W. Hemingway no. 371.78, School of Biology, University of St Andrews (courtesy of Dr Iain Matthews).

**Figure 3.**
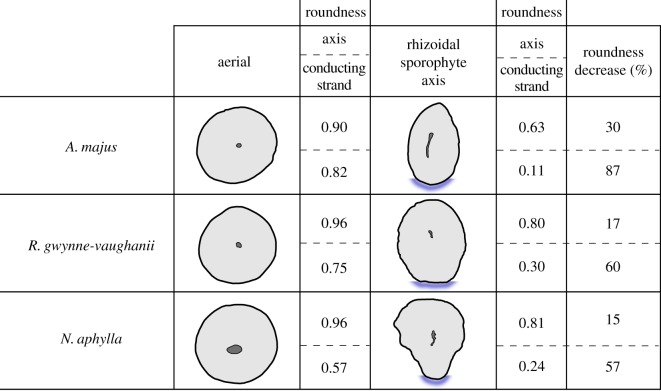
Differences in roundness between orthotropic and plagiotropic axes (rhizoidal sporophyte axes) of Rhynie chert sporophytes. Line drawings based on: *A. majus* orthotropic [31, fig. 14] and rhizoidal sporophyte axes [32, fig. 17] (figure 2*c* this study), *R. gwynne-vaughanii* orthotropic (figure 23, [32]) and rhizoidal sporophyte axes (AGL. Block 22, courtesy of Professor Dianne Edwards) (figure 2*g* this study), *N. aphylla* orthotropic [28, fig. 4.5.A] and rhizoidal sporophyte axes (slide P 2868, courtesy of Professor Hans Kerp) (figure 2*k* this study). Values for roundness for both the axis and conducting strand in orthotropic and rhizoidal sporophyte axes. Roundness quantified using Fiji [30] where a roundness of 1 is a perfect circle. Roundness percentage decrease between the orthotropic and rhizome axis.

The synthesis and reinterpretation of the rooting systems in the Rhynie chert comprised a review of previously published data and examination of specimens in museum collections. These include collections at the Hunterian Museum, University of Glasgow, UK, the School of Earth and Ocean Sciences, Cardiff University, UK and the School of Biology, University of St Andrews, UK. Species assignment was based on the names already assigned in published material. New images of *H. lignieri* were captured from the Rhynie chert slide collection, School of Biology, University of St Andrews using an Olympus BX50 microscope and a Leica M165 FC. Images of *A. majus* from the Kidston collection at the Hunterian Museum, University of Glasgow were captured using a Zeiss 9901 microscope with an attached Nikon Coolpix 4500 camera. To create images of the entire *A. majus* axis and rhizoids (figure 2*g,h*), multiple overlapping photographs were taken and combined to make a single image using AutoStitch [29]. Line drawings of previously published Rhynie chert specimens were made in Inkscape (https://inkscape.org/en/). The roundness of both the axes and conducting strands of *A. majus*, *R. gwynne-vaughnii* and *N. aphylla* was calculated using Fiji [30]. Roundness values were measured from one orthotropic and one rhizoidal axis region for each of the three species examined (figure 3).

## 3. Results

Of the seven documented sporophytes in the Rhynie chert, five are known in sufficient detail to generate complete reconstructions of the structures that carried out rooting functions. *Asteroxylon mackiei* developed rooting organs, referred to as rhizomes [27,33,34]. Rhizomes of *A. mackiei* were radially symmetric axes which branched dichotomously, similar to the roots of extant lycophytes [17]. However, unlike the roots of extant lycophytes [17], absorptive epidermal hairs have not been found on the rhizomes of *A. mackiei* [27,33,34]. The sporophytes of the other four plants for which complete reconstructions have been generated—*H. lignieri*, *A. majus*, *R. gwynne-vaughnii* and *N. aphylla*—comprised a similar level of organization, forming networks of axes with differentiated functions. The vertically growing (orthotropic) shoots bore stomata, suggesting that these axes were photosynthetic, and formed sporangia in either terminal or lateral positions [27,28,31,32,34–37]. Otherwise these vertical axes were naked and did not develop leaves [27,28,31,32,34–37]. The horizontally growing (plagiotropic) axes grew on or through sediment and developed adaptations for rooting function [27,28,31,32,34,36,37]. Here we describe the rooting structures of the sporophytes of the plants of the Rhynie chert. We demonstrate that these specialized rooting axes were bilaterally symmetric and developed unicellular rhizoids from their lower surfaces. This is a unique combination of characters not found in any extant land plants.

### (a) *Aglaophyton majus* rhizoids developed on swellings that formed on axes in contact with the sediment

The *A. majus* sporophyte was a network of branched axes; orthotropic axes growing to a height of approximately 15 cm developed terminal sporangia while rhizoids developed on the surfaces of swellings that formed along the plagiotropic regions of U-shaped portions of axes [27,31,32,37,38] (figure 2*a*,*b*). U-shaped regions of axes were where a single axis had changed growth direction from perpendicular to the ground surface to parallel before returning to perpendicular, forming a U-shaped bend in the axis. The swellings could be up to approximately 6 mm long [37] and developed periodically along the axis. Occasionally neighbouring swellings developed close to each other, forming almost contiguous rhizoid patches [37,38] (figure 2*a*,*b*).

The development of the *A. majus* swellings initiated when part of a radially symmetric plagiotropic axis made contact with the sediment. Subsidiary cells of stomatal complexes dedifferentiated and divided [32]. They divided both periclinally, where the new cell wall is parallel to the rhizome surface, and anticlinally, where the new wall is perpendicular to the rhizome surface [38]. A subset of the resulting cells located on the outside of the rhizome differentiated as rhizoids [37,38] (figure 2*c*,*d*).

After rhizoids initiated on the underside of the plagiotropic axis additional cell divisions developed in the hypodermal layers on the underside of the axis but not on the upper side [37,38]. The cell division and growth on the underside resulted in the formation of a swelling (figure 2*b–d*). Subsequently, conducting tissue differentiated in the hypodermal cells between the central vascular trace and the epidermal rhizoids [37,38]. This conducting tissue grew radially to form one or more conducting traces (figure 2*c*). The combination of the cell division on the underside of the axis and the formation of a radial conducting trace resulted in the formation of a mature structure that was bilaterally symmetric; the side of the axis facing downwards bulged out and was covered with rhizoids while the upward facing axis did not bulge and remained rhizoidless [27,32,37,38] (figure 2*b*–*d*).

### (b) *Rhynia gwynne-vaughanii* developed rhizoids on two distinct structures

The *R. gwynne-vaughanii* sporophyte comprised a network of orthotropic and plagiotropic branching axes [27,31,32,34,36,39]. Orthotropic axes were radially symmetrical and grew to a height of approximately 20 cm [27,31,32,34,36,39] (figure 2*e*). Plagiotropic axes developed rhizoids from two distinct structures [27,31,32,34,36,39] (figure 2*e*,*f*). First, rhizoids developed from the lower epidermis on portions of plagiotropic axes [31,36] (figure 2*g*,*h*). Second, rhizoids developed from multicellular hemispherical projections that protruded from the sides of plagiotropic axes [27,32,36,39] (figure 2*f*). These projections also formed on orthotropic axes where they developed stomata [27,32,36,39]. Vascular tissue did not develop in these hemispherical projections [27,32,36,39].

### (c) *Nothia aphylla* developed rhizoids from a ridge on the lower surface of the rhizome

The *N. aphylla* sporophyte consisted of a below ground plagiotropic axis from which radially symmetrical orthotropic axes grew to a height of approximately 20 cm [27,28] (figure 2*i*). The underside of the rhizome formed a prominent series of ridges—collectively called the rhizoidal ridge—from which rhizoids developed [28] (figure 2*j*). The rhizoidal ridge developed through periclinal and anticlinal divisions in both the dermal layer and hypodermal layers [28]. Rhizoids developed from the outer-most derivative of a periclinal division of dermal cells (figure 2*k*,*l*) [28]. This indicates that a discrete dermal lineage did not exist in the rhizoid-forming zone of *N. aphylla*, as in other Rhynie chert sporophytes. This is unusual, because cell divisions in the dermal lineages that form tip-growing rooting cells (rhizoids or root hairs) in most land plants are anticlinal. Each segment of the ridge (swelling covered with rhizoids on its lower surface) extended longitudinally for up to 10 mm along the lower surface of the rhizome [28]. Almost all epidermal cells along this ridge developed rhizoids; there were few or no cells that differentiated as rhizoidless epidermal cells [28] (figure 2*j*–*l*). The region that developed rhizoids was connected to the central vascular strand by a bridge of vascular tissue termed the connective [28] (figure 2*k*). The connective can be distinguished from the surrounding cortex by the presence of thick walled cells which extend from the vascular trace towards the rhizoid-developing epidermis and the absence of intercellular spaces [28] (figure 2*k*).

### (d) The tuberous rhizome axis of *Horneophyton lignieri* developed rhizoids

The *H. lignieri* sporophyte comprised orthotropic axes terminating in sporangia that grew to a height of approximately 20 cm and a partially buried tuberous rhizome [27,31,34,35] (figure 2*m*,*n*). The rooting structure consisted of the swollen and sometimes branched rhizome which developed a dense covering of rhizoids in patches from the lower surface [6,27,31,34,40] (figure 2*n*–*p*). The internal structure of these swellings was rich in parenchyma. Epidermal cells on these swellings were small and box-shaped and each differentiated as a rhizoid (figure 2*p*) [31]. Orthotropic axes were radially symmetric, developed a central conducting strand and grew vertically from the rhizome. In contrast to the orthotropic axes the rhizome lacked a central conducting strand. The base of the conducting strands from orthotropic axes terminated within the rhizome and was marked by regions of brown-celled parenchyma [31].

### (e) Rhizoid-bearing regions of Rhynie chert sporophyte axes developed bilateral symmetry

**Figure 4.**
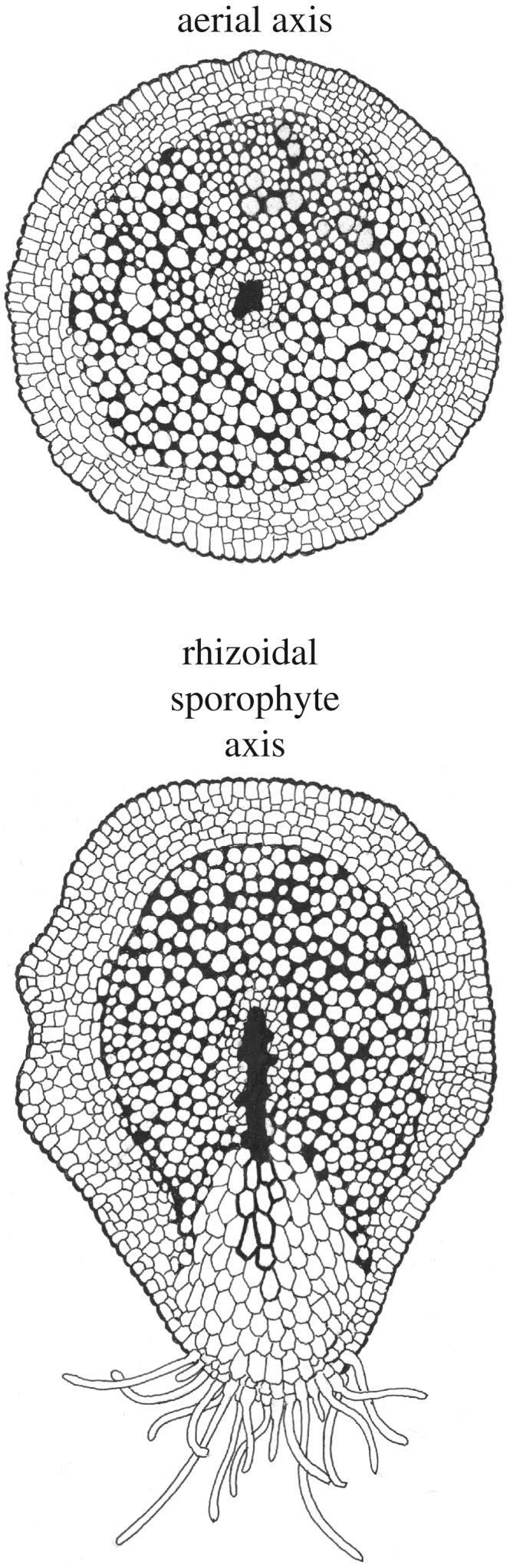
Schematic showing an aerial (orthotropic) axis and a rhizoidal sporophyte axis (plagotropic axis) when viewed in transverse section. The aerial axis is radially symmetric. Tissues are arranged in concentric rings: epidermis, hypodermis, cortex (large intercellular air spaces in the cortex are highlighted in solid black) with the conducting strand at the centre. The rhizoidal sporophyte axis is bilaterally symmetric with rhizoids on the underside. The tissues are distended towards the lower side of the axis and not arranged in concentric rings as in the aerial axis. A region of tissue extends from the conducting strand to the rhizoid-bearing epidermis on the lower side of the axis. This tissue comprises larger cells than elsewhere in the section. The walls of eight cells in this section are thicker than the others. These cells form a line from the vascular trace to the lower side of the axis. The schematics are based on transverse sections of *N. aphylla* from reference [28], aerial [28, fig. 4.5.A] and rhizoidal sporophyte axes (slide P 2868, courtesy of Professor Hans Kerp) (figure 2*k* this study).

The rooting structures of *A. majus*, *R. gwynne-vaughnii*, *N. aphylla* and *H. lignieri* sporophytes consisted of stretches of plagiotropic axes with additional tissue differentiation associated with rhizoid development compared with the surrounding regions of the axes. Furthermore, rhizoids most often developed from the lower surfaces. The tissue differentiation associated with the development of rhizoids led to the formation of axes that were bilaterally symmetric compared with the radially symmetric orthotropic axes. This difference between rhizoid-forming regions and radial orthotropic axes is seen most clearly in *H. lignieri*. *H. lignieri* developed radially symmetric orthotropic axes with central radially symmetric conducting strands. By contrast the rhizome was tuberous and lacked a central conducting strand, and the lower surface was covered in rhizoids which developed from small box-shaped cells. The rhizoidal sporophyte region of *H. lignieri* was markedly different from the orthotropic axes and developed bilateral symmetry owing to the development of rhizoids from its base. Although the rhizoidal sporophyte regions of the other three species—*A. majus*, *R. gwynne-vaughnii* and *N. aphylla*—were more similar to their orthotropic axes than *H. lignieri* they all developed bilaterally symmetric rhizoidal sporophyte axes that were quantitatively different from their orthotropic axes. We carried out quantitative analysis of the shape of rooting axes and individual conducting strands when viewed in transverse section. A measure of roundness was calculated using the equation (*R* = 4*A*/(*πL*^2^)) [30], where *A* is the cross-sectional area in transverse section, and *L* is the length of the longest axis of the transverse section [30]. A value of *R* = 1 indicates a perfect circle (symmetric) and values less than 1 are less round (asymmetric) [30]. Orthotropic sporophyte axes of *A. majus*, *R. gwynne-vaughnii* and *N. aphylla* were relatively symmetric, with roundness values of 0.90, 0.96 and 0.96, respectively (figure 3). By contrast the rhizoid-bearing axes are less symmetric and less round; roundness values were 0.63, 0.80 and 0.81 for *A. majus*, *R. gwynne-vaughnii* and *N. aphylla*, respectively (figure 3). Rhizoid-bearing axes were 30, 17 and 15% less round than in orthotropic axes in these species, respectively (figure 3). The difference in symmetry between the orthotropic and plagiotropic axes in each species was even more pronounced in the conducting strands. *A. majus*, *R. gwynne-vaughnii* and *N. aphylla* developed radially symmetric (round) conducting strands in orthotropic axes (figure 3). Conducting strand roundness was 0.90, 0.96 and 0.96 in *A. majus*, *R. gwynne-vaughnii* and *N. aphylla*, respectively (figure 3). By contrast, conducting strand roundness was 0.11, 0.30 and 0.24 in *A. majus*, *R. gwynne-vaughnii* and *N. aphylla*, respectively (figure 3). The conducting strand was elongated in the direction of the surface where rhizoids developed and this accounted for the low roundness values (figure 3, blue shading highlights the rhizoid-developing surface). Therefore, the conducting strands of *A. majus*, *R. gwynne-vaughnii* and *N. aphylla* were 87, 60 and 57% less round in the plagiotropic rhizoid-bearing axis than in orthotropic axes (figure 3). These data indicate that the rooting axes of *H. lignieri*, *A. majus*, *R. gwynne-vaughnii* and *N. aphylla* were distinguished from orthotropic axes by the transverse sectional shape of both their axes and conducting strands. The development of rooting systems of these sporophytes, therefore, involved the transition from radially symmetrical axes to bilaterally symmetrical axes, with rhizoids developing on the lower surface of the bilaterally symmetrical axes (figure 4). The combination of these traits—rhizoids on bilaterally symmetric sporophyte shoot axes—is unique among land plants. The phylogenetic position of these species suggests that rhizoidal sporophyte axes represented the ancestral rooting structure among the vascular plants.

## 4. Discussion and conclusion

The development of the rhizoidal sporophyte axes of *H. lignieri*, *A. majus*, *R. gwynne-vaughnii* and *N. aphylla* comprised a unique combination of characteristics; unicellular rhizoids developed on portions of the lower surface of bilaterally symmetric plagiotropic shoot axes (figures 2 and 4). The phylogenetic relationships of *H. ligneri* and *A. majus* (protracheophytes), *R. gwynne-vaughanii* (an early diverging vascular plant) and *N. aphylla* (an early diverging lycophyte) indicate that these species span the origin of the vascular plant lineage [2] (figure 1). Based on the development of rhizoidal sporophyte axes in these taxa and their phylogenetic position we hypothesize that rhizoidal sporophyte axes constituted the structures that carried out the rooting function in the first vascular plants. We propose that rhizoidal sporophyte axes represent a third major type of rooting system (figure 2). Rhizoidal sporophyte axes are distinct from sporophytic roots of extant vascular plants; roots are radially symmetric, develop from a root meristem, form an apical root cap and grow root hairs from the epidermis over their entire circumference [1,5]. Rhizoidal sporophyte axes are also different from all gametophytic rhizoid-based rooting systems [1,6] because rhizoidal sporophyte axes developed in the sporophyte. They represent a solution to carrying out the rooting function in free-living sporophytes before the evolution of sporophytic roots—axes with an apical meristem covered in a root cap—in the lycophyte and euphyllophyte lineages. All species with this organization of rooting structure are now extinct.

Although all species developing rhizoidal sporophyte axes are now extinct, a handful of extant taxa evolved modified shoots that carry out rooting functions following the loss of roots during evolution [18,41]. For example, the sporophytes of all Psilotaceae and some members of the Hymenophyllaceae ferns are rootless and shoots carry out rooting function. These modified shoots develop multicellular hairs (trichomes) that may carry out similar functions to the unicellular rhizoids and root hairs of other tracheophytes [2,42,43]. Given their functional similarity, these multicellular hairs have been termed rhizoids in the Psilotaceae [42,44]. The modified shoot axes of the Psilotaceae and Hymenophyllaceae are different from the extinct rhizoidal sporophyte axes of the Rhynie chert plants reported here in at least two ways. First, the hairs of these species are multicellular and develop over the entire surface of a radial shoot axis [2,42,43]. Second, these shoots are radially symmetric; the conducting strand and internal tissues—which include an endodermis in the Psilotaceae, a tissue absent from the rhizoidal sporophyte axes in the Rhynie chert [27,42,44]—of these modified shoot axes are arranged in a radial organization and absorbent hairs develop over the entire circumference of the axes giving them radial symmetry. Shoots modified to carry out the rooting function also develop in some lycophytes [45–47]. Hair-bearing protocorms are tuberous and present in a number of members of the Lycopodiales, including *Lycopodium cernuum* [47] and *Phylloglossum drummondii* [48], and resemble the rhizoid-bearing rhizome of *H. lignieri*. However, the hairs on these extant lycophyte protocorms are frequently multicellular [45,46], unlike the unicellular rhizoids on rhizoidal sporophyte axes of the Rhynie chert. Therefore, similar structures—shoot axes bearing multicellular, tubular hairs—evolved independently in rootless lineages of extant tracheophytes in which shoots were modified to carry out rooting function.

The evolution of rhizoidal sporophyte axes in the common ancestor of vascular plants will have had a number of physiological and ecological impacts. First, the combination of rhizoids and the modification of internal conducting tissues would have enhanced water and nutrient uptake into the transpiration stream. The large number of rhizoids would have provided a relatively large surface area over which water and inorganic nutrients were taken up and delivered to the vascular system for transport to the rest of the plant. This would have contributed to the nutritional independence of the free-living sporophyte. Second, the masses of rhizoids produced on these structures would have anchored the plants to the sediment and acted as an interface for the interaction between the plant and the soil microflora. The enhanced interaction with the sediment and increased transpiration will have had dramatic impacts on nutrient and water cycles just before the radiation of the vascular plants.

We conclude that bilaterally symmetric axes bearing rhizoids were the sporophyte rooting structures of *H. lignieri*, *A. majus*, *R. gwynne-vaughnii* and *N. aphylla* and we suggest that these structures represents the land plant rooting system that existed in the common ancestor of vascular plants.

## Data accessibility

This article has no additional data.
